# Locally advanced duodenal gangliocytic paraganglioma treated with adjuvant radiation therapy: case report and review of the literature

**DOI:** 10.1186/1477-7819-3-15

**Published:** 2005-03-01

**Authors:** Adrian Wong, Alexander R Miller, John Metter, Charles R Thomas

**Affiliations:** 1 Department of Radiation Oncology, School of Medicine, University of Texas Health Science Center @ San Antonio, San Antonio, TX, 78229, USA; 2 Division of Surgical Oncology, Cancer Therapy & Research Center, San Antonio, TX, 78229, USA; 3 Department of Pathology, Methodist Hospital, San Antonio, TX, 78229, USA

## Abstract

**Background:**

Gangliocytic paraganglioma are rare neoplasms that predominantly arise in periampulary region. Though considered benign the disease can spread to regional lymphatics.

**Case presentation:**

A 49 year old woman presented with melena and was found to have a periampullary mass. Endoscopic evaluation and biopsy demonstrated a periampullary paraganglioma. The tumor was resected with pylorus-preserving pancreaticoduodenectomy and was found to represent a gangliocytic paraganglioma associated with nodal metastases. In a controversial decision, the patient was treated with adjuvant external beam radiation therapy. She is alive and well one year following resection. The authors have reviewed the current literature pertaining to this entity and have discussed the biologic behavior of the tumor as well as the rationale for treatment strategies employed.

**Conclusion:**

Paraganglioma is a rare tumor that typically resides in the gastrointestinal tract and demonstrates low malignant potential. Due to rarity of the disease there is no consensus on the adjuvant treatment even though nearly 5% of the lesions demonstrate the malignant potential.

## Background

Gangliocytic paragangliomas are unusual neoplasms that may be identified anywhere within the gastrointestinal tract, but predominate in the periampullary region. This entity was first discussed by Dahl *et al *in 1957, and subsequently reported by Taylor and Helwig in 1962 [1,2]. Kepes and Zacharias named the tumor and described its characteristics in 1971 [3]. The pathognomonic features of these neoplasm is the identification of three distinct cellular elements: spindle cells, epithelial cells, and ganglion cells. These tumors are considered benign, yet occasionally metastasize to regional lymph nodes, as well as to distant organs [4]. Long term survival is common with appropriate resection. We report a case of a 49-year-old female who presented with melena and was found to have a periampullary gangliocytic duodenal paraganglioma. The details of the clinical presentation, histopathological findings, and therapeutic choices are provided.

## Case presentation

**Figure 1:**
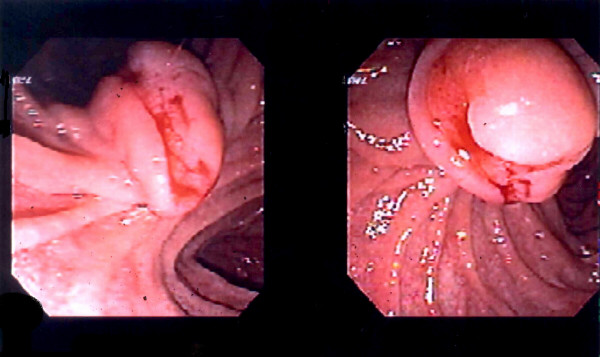
Endoscopic photograph of the paraganglioma demonstrating an exophytic tumor whose edges enfold around a central area of necrosis that is actively bleeding.

A 49-year-old woman presented with a 6-month history of melena. During the preliminary consultation, she also complained of right upper quadrant pain, which radiated to her right lower quadrant and upper back. She underwent upper gastrointestinal (GI) endoscopy which demonstrated a 3 cm, ulcerated, ampullary mass (figure 1). Endoscopic biopsies suggested the diagnosis of paraganglioma. The lesion did not obstruct the ampullary orifice. Both computed tomography (CT) and magnetic resonance imaging (MRI) of the abdomen failed to demonstrate this lesion or any additional abnormalities.

The patient underwent pylorus-preserving pancreaticoduodenectomy and lymph node dissection. The postoperative course was uneventful except for delayed gastric emptying diagnosed by frequent vomiting during postoperative days 7 to 10, and confirmed by gastrograffin swallow. These symptoms eventually resolved with conservative management and the patient was discharged from the hospital on 15^th ^postoperative day.

### Histopathological analysis

Gross pathological evaluation of the resected specimen included a portion of duodenum with ampulla, measuring 16 cm. in length, and a portion of pancreatic head measuring 5 cm. in length. There was a polypoid periampullary mass protruding into the duodenal lumen, measuring 1.4 × 1.2 × 0.7 cm. On cut section, the mass was moderately firm and pink-tan. It was circumscribed but unencapsulated, and appeared to be covered by normal appearing duodenal mucosa. There was no evidence of pancreatic invasion by the tumor. A total of seven lymph nodes were also removed, 5 peripancreatic (3.0 cm in greatest dimension) and 2 periduodenal (1.2 cm and 1.0 cm in greatest dimension, respectively).

**Figure 2:**
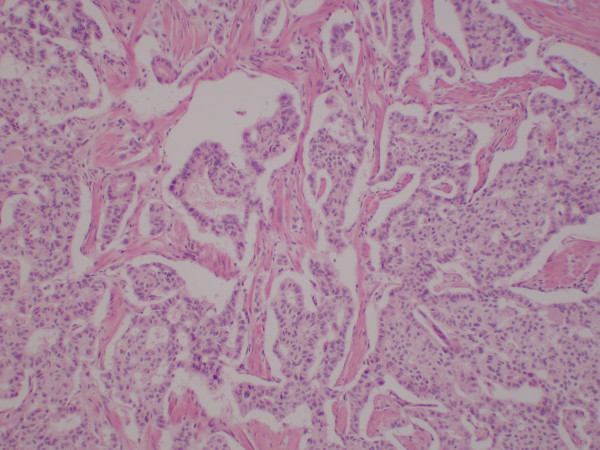
Photomicrograph showing areas of the tumor that had an epithelial pattern resembling carcinoid tumor or islet cell tumor. These cells were cohesive, uniform in size and shape with small round uniform nuclei, and formed rosettes, cribriform structures, solid nests and trabecular cords (Hematoxylin and Eosin original magnification ×100).

**Figure 3:**
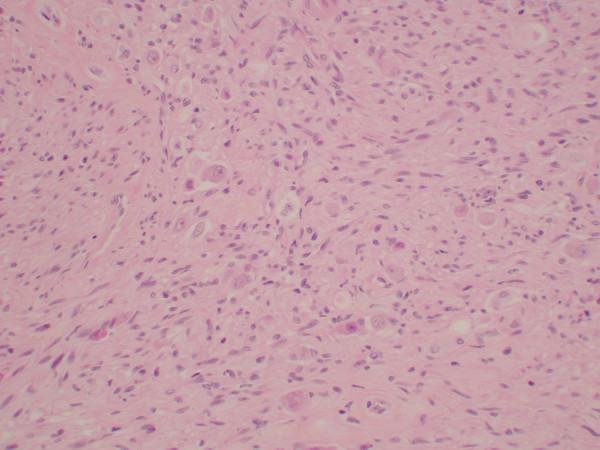
Photomicrograph showing areas of the tumor that consisted of a proliferation of spindled neurofibrillary cells with admixed larger polygonal ganglionic cells (Hematoxylin and Eosin original magnification ×100).

**Figure 4:**
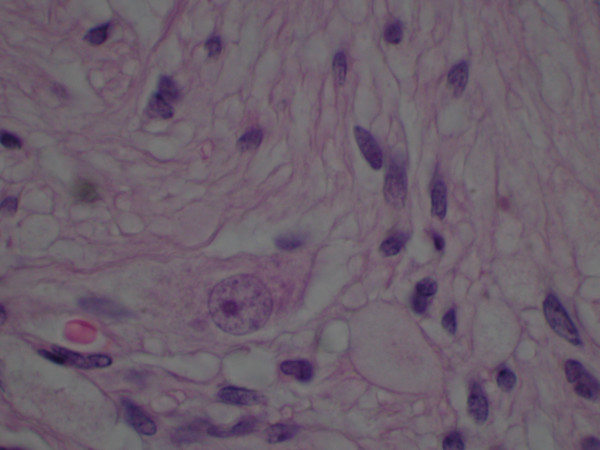
Photomicrograph showing the ganglionic cells having large round nuclei with chromatin clearing and large central nucleoli. The cytoplasm is abundant and amphophilic staining. The ganglionic cells are admixed with the spindled cells (Hematoxylin and Eosin original magnification ×400).

**Figure 5:**
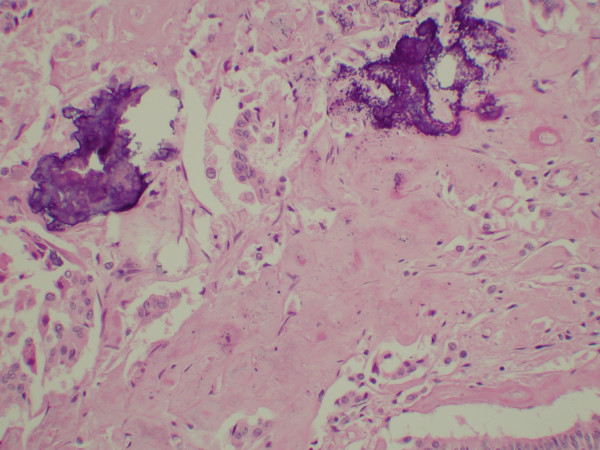
Photomicrograph showing the focal areas of stromal hyalinization with calcification. Special stains for amyloid were negative (Hematoxylin and Eosin original magnification ×200).

Histologically, the tumor consisted of a complex neoplastic proliferation that included a component resembling carcinoid or islet cell tumor (figure 2), admixed with a proliferation of spindled neurofibrillary cells and larger polygonal cells demonstrating gangliocytic differentiation (figures 3 and 4). There were areas of stromal hyalinization resembling amyloid, with focal calcification (figure 5). Congo red and Thioflavin T stains were negative for amyloid. The tumor extended through the muscularis propria and along the common bile duct, but did not invade the pancreas. The resection margins were free of tumor. Metastatic paraganglioma was present in 6 of 7 periduodenal and peripancreatic lymph nodes. The metastatic lymph nodes showed the same mixed histologic features as the primary tumor.

Immunohistochemical analysis demonstrated that the tumor stained positively for S-100, chromogranin, synaptophysin, and cytokeratin AE1 and AE3. No reactivity was observed with MART-1 or HMB 45. Staining for c-kit (CD 117) was performed on sections of the primary tumor and of one of the lymph nodes with metastatic tumor. The carcinoid-like epithelial cells and the spindle-shaped neurofibrillar cells stained negatively for the cell marker c-Kit. The gangliocytic cells stained strongly positive for c-kit.

### Adjuvant therapy

Due to the evidence of regional lymph node metastasis the patient was counseled regarding adjuvant therapeutic options. The treating physicians queried recognized experts in the field of radiation therapy for gastrointestinal malignancies via email and a consensus developed that external beam radiation therapy might be reasonable, although it was admitted that no data are available regarding the use of this modality for this disease entity. Ultimately, a decision was made to administer external beam radiotherapy to the abdomen in an effort to eradicate any possible residual disease not removed during surgery and to reduce the risk of locally recurrent disease. No chemotherapy was advised due to the rarity of distant metastases and the lack of response of these neoplasms to conventional systemic therapy.

The patient was treated with intensity-modulated radiotherapy in 28 fractions of 180 cGy/fraction over 37 elapsed days; 6 mv photon beam energy was used. The target was a postoperative tumor bed with a 5–10 mm margin. The total dose was 5,040 cGy. She tolerated treatment well and is now symptom free more than one year following resection. Surveillance CT scans and endoscopy have been performed both of which reveal no evidence of recurrent disease.

## Discussion

Gangliocytic paraganglioma is a rare, typically benign tumor of the gastrointestinal tract most commonly located in the second portion of the duodenum, with a few cases having involved the jejunum and pylorus [5-7]. Burke *et al *reported that there seems to be a slight male predominance and an average age of 54 at presentation [5]. Other authors have denied that there is any gender preference [8]. This lesion usually presents with abdominal pain and gastrointestinal bleeding due to mucosal ulceration. Obstructive jaundice is less common [9].

Histologically, our patient's tumor demonstrated the characteristic tricellular pattern of gangliocytic paragangliomas. These tumors are typically composed of an admixture of ganglion cells, spindle cells and epithelial cells [5,10-13]. These tumors are submucosal, and rarely recur or metastasize [14-16]. In most reported cases regional lymph node involvement, the metastatic cells consist predominantly of epithelial cells [15]. A relatively unique element of the case we present is that six of 7 regional lymph node metastases contained all three characteristic cell types, and thus, demonstrated the possibility for each of these cell types to acquire a malignant potential.

Immunohistochemically these tumors stain positive for a variety of markers as was demonstrated in this report. Such markers include those mentioned above as well as neuron-specific enolase, pancreatic polypeptide, somatostatin, myelin basic protein and neurofilament proteins [6,12,13,17]. The origin of gangliocytic paragangliomas has been widely debated and includes hypotheses ranging from a hamartomatous derivation to cellular elements arising from pancreatic neuroendocrine tissue, or that of the retroperitoneal celiac sympathetic or parasympathetic plexuses [4,12].

There is no data in the literature to guide clinicians on the use of adjuvant therapy despite the fact that approximately 5% of cases demonstrate malignant features [4]. Since this patient had multiple positive lymph nodes and is relatively young, a trial of adjuvant radiotherapy to the operative bed was considered reasonable and was endorsed by radiation oncologists at high volume cancer centers queried via email.

## Conclusion

Gangliocytic paraganglioma is a rare duodenal tumor that can present with non-specific symptoms. Positive diagnosis can be obtained histologically by observing three characteristic cell types. Although this tumor is considered benign, the possibility exists for regional lymph nodal spread. Due to the rarity of the disease, no clear adjuvant treatment strategy has been determined in cases that demonstrate regional or distant metastasis.

## Competing interests

The authors(s) declare that they have no competing interests.

## Authors' contributions

AW wrote the original manuscript.

AM performed surgical resection and prepared requested revisions of the manuscript.

JM performed histopathological evaluation of the lesion and prepared photomicrographs.

CT administered radiation therapy to the patient, made editorial suggestions, and supervised AW.
